# Stemness signature RBBP7 reprograms the immune microenvironment to inform a prognostic model in esophageal carcinoma

**DOI:** 10.3389/fimmu.2026.1789900

**Published:** 2026-05-19

**Authors:** Yubing Liu, Xiao Yang, Ruiqin Du, Lanxiang Wu, Qingchen Wu

**Affiliations:** 1 Department of Cardiothoracic Surgery, The First Affiliated Hospital of Chongqing Medical University, Chongqing, China; 2 Pharmacogenetics and Pharmacogenomics Laboratory, School of Pharmacy, Chongqing Medical University, Chongqing, China

**Keywords:** esophageal carcinoma, esophageal neoplasms, prognosis, single cell RNA sequencing, T follicular helper cell, tumor microenvironment, tumor stemness

## Abstract

**Background:**

Esophageal carcinoma has high mortality and poor prognosis. Current multimodal therapies remain limited by scarce actionable targets and suboptimal systemic efficacy. Tumor stemness programs sustain invasive, therapy-resistant cells and may offer new opportunities for precision stratification and treatment.

**Methods:**

The present study has demonstrated the multifaceted roles of stemness genes in esophageal cancer through integrated multi-omics research. Firstly, the analysis of bulk RNA-seq revealed dysregulation of stemness genes in cancer. Utilizing the high-dimensional WGCNA approach in the context of single cell RNA-seq, we have successfully identified modules that are associated with tumor stemness. Subsequently, Cox regression and LASSO analysis were employed to identify prognostic genes and construct a predictive model. CellChat and functional enrichment studies explored crosstalk between model genes and the microenvironment, while multiple experiments validated the efficacy of these model genes.

**Results:**

Through multimodal analysis, stemness exhibits significant differences between esophageal cancer and adjacent normal tissue. By integrating multiple algorithms, we constructed a stemness gene prognostic model. This model accurately predicts prognosis and drug response, with its 1-, 2-, and 3-year survival predictions outperforming TNM staging. The model gene RBBP7 emerges as the most influential prognostic factor. Its overexpression mediates heightened tumor cell stemness and correlated with T follicular helper cells infiltration, thereby reshaping the tumor microenvironment.

**Conclusion:**

The stemness gene model has been demonstrated to possess the capacity to accurately predict the prognosis of patients diagnosed with esophageal cancer. It is noteworthy that the model gene RBBP7 has been identified as a promising therapeutic target for addressing the issue of stemness in esophageal cancer.

## 1 Introduction

Esophageal carcinoma (ESCA) is a highly aggressive malignancy of the digestive tract, with increasing global incidence and mortality rates. Epidemiological studies indicate that ESCA remains a leading cause of cancer-related deaths in the United States, imposing a substantial disease burden (1). The insidious nature of early-stage disease often results in delayed diagnosis, with many patients presenting at advanced stages. Although surgery, radiotherapy, and chemotherapy are routinely applied, overall survival remains unsatisfactory, partly due to the lack of effective targeted agents and the limited benefit of current immunotherapeutic approaches (2). Therefore, elucidating the molecular mechanisms underlying esophageal cancer progression and identifying robust prognostic biomarkers are of considerable clinical significance.

Current research on esophageal cancer has largely focused on pathological mechanisms and therapeutic strategies. Established risk factors include smoking, alcohol consumption, and chronic gastroesophageal reflux (3). In recent years, increasing attention has been directed toward cancer stem cells and tumor stemness, as stemness-associated phenotypes are closely linked to aggressiveness, therapeutic resistance, and recurrence, thereby substantially influencing patient outcomes (3). However, studies systematically interrogating specific stemness-related molecular determinants remain limited, particularly with respect to early diagnosis and prognostic assessment in esophageal cancer.

Against this background, we investigated the role of the stemness-associated gene retinoblastoma-binding protein 7 (RBBP7) in esophageal cancer. RBBP7 has been implicated in the regulation of tumor stemness and may play a critical role in esophageal tumorigenesis and progression (4). Prior studies have reported that elevated RBBP7 expression is associated with unfavorable prognosis across multiple cancer types, underscoring its biological relevance (5). Nevertheless, comprehensive evidence regarding the contribution of RBBP7 to the maintenance of stemness features in ESCA remains insufficient.

In the present study, we combined bioinformatics analyses with experimental validation. Using publicly available datasets and transcriptomic profiling, we established an esophageal carcinoma stemness-related model (ECSM) and demonstrated that it achieved improved predictive performance for postoperative outcomes compared with conventional TNM staging. We further identified RBBP7 as the most influential high-risk gene among the ECSM weighted features and experimentally verified that RBBP7 promotes ESCA cell invasion and the maintenance of stemness-associated properties. Additionally, elevated RBBP7 expression was found to enhance the infiltration of T follicular helper cells, thereby suppressing the immune microenvironment. Collectively, these findings suggest that RBBP7 holds substantial potential as a prognostic biomarker and a candidate therapeutic target, and our work provides new insights into the molecular basis and clinical management of esophageal cancer.

## 2 Materials and methods

### 2.1 Data collection and preprocessing

We secured bulk RNA sequencing (RNA-seq) data, paired with corresponding survival and clinical details, directly from the TCGA repository (https://www.cancer.gov/ccg/research/genome-sequencing/tcga) employing the TCGAbiolinks R package (6). The validation set was obtained from Gene Expression Omnibus (GEO) database (https://www.ncbi.nlm.nih.gov/geo/) (7). The expression matrix was subsequently combined and transformed into transcripts per million (TPM) units. Annotated single-cell RNA sequencing (scRNA-seq) information pertaining to esophageal carcinoma (GSE160269) was sourced from the Gene Expression Omnibus (GEO) database (https://www.ncbi.nlm.nih.gov/geo/) (8). This dataset underwent rigorous processing through the Seurat pipeline (9). These include the following annotated genes: COL1A1 (fibroblasts), EPCAM (epithelial cells), VWF (endothelial cells), CD3D (T cells), and others. Following quality control, log_2_ transformation, feature selection (2000 highly variable genes), and scaling, we initially performed dimensionality reduction using principal component analysis (PCA), followed by additional compression via Uniform Manifold Approximation and Projection (UMAP) to construct the final integrated dataset. The stemness genes were downloaded via StemChecker (10).

### 2.2 Stemness assessment

The R package synapser is used to assess stemness in bulk RNA sequencing. Inputting the integrated TPM and DNA methylation matrices yields the mRNA expression- based stemness indices (mRNAsi) and DNA methylation-based stemness indices (mDNAsi), respectively (11). Stem cell potential in scRNA-seq data is evaluated viathe CytoTRACE R package. Stemness scores are generated for each cell by inputting the integrated scRNA-seq expression matrix.

### 2.3 Construction of the stemness prognostic model

To obtain a more precise esophageal carcinoma stemness model (ECSM), tumor cells in epithelial cells from single-cell data were annotated using inferCNV (12). In the subsequent stage of the experiment, the stemness of epithelial cells was evaluated using CytoTRACE (13), and stemness-related gene modules were identified via high-dimensional WGCNA (hdWGCNA) (14). The module genes that demonstrated the strongest correlation with stemness scores in tumor cells were retained. Following this, single-factor Cox regression assessed module gene expression in bulk RNA-seq data, with the aim of identifying hub genes that influence prognosis. The remaining genes underwent differential expression analysis of cancerous versus healthy epithelial cells in single-cell data, retaining solely genes exhibiting expression variations. Subsequent to the optimization of the residual differential genes by means of LASSO regression, an esophageal cancer stemness model was constructed via multivariate Cox analysis.

### 2.4 Pathway enrichment and microenvironment infiltration analysis

Differential analysis between groups was performed using Limma on the bulk RNA-seq data (15). For the single-cell data, the FindAllMarkers function in Seurat was used to identify specific genes of cell clusters. The genes underwent pathway enrichment analysis using clusterProfiler in R (16). For immune characterization, the IOBR package integrated multiple algorithms to analyze bulk RNA-seq data (17). For scRNA-seq, immune cells were annotated using markers prior to analysis, and cell-to-cell engagements were assessed through the utilization of the CellChat R package (18).

### 2.5 Cell culture

The esophageal cancer cell lines, including HET, TE1, TE10, ECA109, ECA150, and ECA510, were cultured in DMEM enriched with 10% fetal bovine serum, penicillin at a concentration of 10,000 U/mL, and streptomillin at 10 μg/mL. These cells were kept under standard conditions at 37 degrees Celsius in an atmosphere containing 5% carbon dioxide.

### 2.6 RNA Interference-Mediated Knockdown of RBBP7

The transient knockdown (KD) of human RBBP7 was achieved through the use of sequence-specific siRNAs (Sangon Biotech, Shanghai) and Lipofectamine RNAiMAX reagent (Invitrogen, 13778075). Three siRNA sequences of RBBP7 were compared (**Supplementary Table 1**). A scrambled siRNA sequence served as the negative control. Cells demonstrating successful knockdown were subsequently utilized for further experimental procedures.

### 2.7 RNA extraction and qRT-PCR

We extracted total RNA using Trizol reagent from Thermo Fisher Scientific, then converted it into complementary DNA (cDNA) via reverse transcription with the PrimeScript First Strand cDNA Synthesis Kit manufactured by Takara in Shiga, Japan. The quantification of target gene mRNA expression was conducted through quantitative PCR analysis utilizing SYBR Green Premix Ex Taq (Takara). The thermal cycling protocol consisted of an initial denaturation at 95 °C for 30 seconds, followed by 40 amplification cycles (95 °C for 10 seconds, 52 °C for 10 seconds, and 72 °C for 10 seconds). Gene expression levels were subsequently normalized against the endogenous reference gene GAPDH for relative quantification. The primers utilized are outlined in **Supplementary Table 2**. Gene expression levels were quantified through the 2−ΔΔCt analytical method.

### 2.8 Western blot

We extracted proteins with RIPA Lysis Buffer (Thermo Fisher Scientific), followed by electrophoretic separation on 10% SDS-polyacrylamide gels. Following the transfer of the membranes to PVDF, a solution of 5% non-fat milk was employed to block them at ambient temperature for a period of one hour. Following this step, the membranes were treated with specific primary antibodies - either anti-RBBP7 (Cat20365-1-AP, Proteintech, China) or anti-GAPDH (Cat60004-1-Ig, Proteintech, China) - and maintained at 4 °C for overnight incubation. Subsequently, the samples were incubated with the appropriate horseradish peroxidase (HRP)-conjugated secondary antibody (Invitrogen) at room temperature for one hour. The proteins were visualized employing Bio-Rad system (Hercules, USA). The intensity was quantified through grayscale analysis through manually adjust the gray values of the background area, and finally obtain the optical density values for quantitative analysis using ImageJ software.

### 2.9 Sphere formation assay

Cells were isolated and sseeded in 24-well non-adherent plates (Corning, 3473). The maintenance of cells was conducted in serum-free DMEM/F12 medium (Gibco, C11330500BT), which was supplemented with 20 ng/mL epidermal growth factor (Sigma, E9644), 1% methylcellulose (R&D Systems, HSC001), 20 μg/mL B27 supplement (Gibco, 17504044), and 20 ng/mL basic fibroblast growth factor (Peprotech, 100-18B). The culture medium was refreshed at 48-hour intervals in order to maintain optimal growth conditions. The plates were then subjected to an incubation process at a temperature of 37 °C within a controlled environment containing 5% CO_2_ for a period of approximately 14 days. This was undertaken until the observation of spheroid formation. Spheroids images were then put into ImageJ to count spheres with a diameter greater than 50 μm.

### 2.10 Extreme limiting dilution assay

The cells were cultivated in 96-well ultra-low attachment plates (Corning, 3474) using the sphere culture medium that had been previously described. Cell seeding densities were set at 7, 15, 30, 60, 120, and 250 cells per well, with 24 replicate wells prepared for each density. Subsequent to a 7-day incubation period, the number of wells containing spheres for each cell density group was recorded. The subsequent analysis of this data was conducted using the online ELDA statistical tool (19).

### 2.11 Transwell migration and invasion assay

The assessment of cell migration and invasion capabilities was conducted utilising Boyden chamber systems, which were equipped with Transwell membrane filter inserts (Cat. No. 3422, Corning Costar). In summary, for the migration assay, 5 x 10^4^ cells were plated into 24-well Transwell chambers (8 μm pore size), while for the invasion assay, cells were seeded into Matrigel-coated chambers. Both assays were conducted in complete medium, which was supplemented with 10% foetal bovine serum (FBS). The incubation periods for migration and invasion were set at 24 hours and 48 hours, respectively. Subsequent to the process of incubation, non-migratory or non-invasive cells that remained on the upper surface of the membrane were meticulously removed. The cells that had traversed the membrane were then fixed and stained with 0.4% crystal violet. Quantitative analysis was performed by enumerating the stained cells from five randomly selected fields per chamber under a light microscope. We use ImageJ to In Image to convert the images to 8-bit grayscale, adjust the threshold, perform cell segmentation, and generate the number of cells for statistics. The results are expressed as the mean ± standard error (SE) from three independent replicates.

### 2.12 Mouse ESCA model

Female C57BL/6J mice were procured from GemPharmatech (Nanjing, China) and maintained in a specific pathogen-free (SPF) facility. The animals, aged between five and seven weeks at the commencement of the experiment, were housed under controlled environmental conditions (24 ± 2 °C, 50 ± 10% relative humidity) with a standardized 12-hour photoperiod (08:00-20:00 light phase). It is noteworthy that throughout the duration of the study, all mice were granted unmitigated access to sustenance and water. The experimental protocol was reviewed and granted ethical approval by the Institutional Animal Care and Use Committee of The First Affiliated Hospital of Chongqing Medical University (Pro No. 2024-075-01).

In order to induce the formation of tumors, AKR cell suspensions were prepared at a density of 1×10^5^ cells in a 1:1 mixture of phosphate-buffered saline (PBS) and Matrigel matrix (Corning, 354230). A total volume of 100 μL (comprising 50 μL Matrigel and 50 μL PBS) containing the tumor cells was administered via subcutaneous injection into the flank region of each mouse. Tumor growth was monitored by caliper measurements of the vertical diameter at 48-hour intervals. When tumors reached the predetermined ethical size limit, animals were humanely euthanized and the xenograft tumors were excised for photographic documentation.

### 2.13 Immunohistochemical and immunofluorescence staining

Totally 53 patients were enrolled in this study. Tissue samples were embedded in paraffin, sectioned, and processed through standard deparaffinization and rehydration steps. Antigen unmasking was conducted in a citrate buffer (pH 6.5) with microwave heating for a duration of three minutes. The study found that endogenous peroxidase activity was effectively inhibited by treatment with a 3% hydrogen peroxide solution, followed by blocking with 10% normal goat serum. Sections of tissue were then subjected to incubation with an anti-RBBP7 primary antibody (1:400 dilution, Proteintech, 84330-1-RR) at 4 °C for a period of 16 hours. The detection stage of the process was performed using HRP-conjugated secondary antibodies (IHC kit, ZSBIO, pv-9002), following an incubation period of 60 minutes. The chromogenic visualization was achieved through the application of DAB substrate (ZSGB-BIO, ZLI-9018), with the reaction terminated by rinsing with distilled water. The process of nuclear counterstaining was executed by applying hematoxylin (Coolaber, SL7050) for a duration of 4–6 minutes, followed by a thorough washing procedure. Sections were then dehydrated through a series of graded alcohols, cleared in xylene, and permanently mounted with neutral balsam.

In the context of multiplex immunofluorescence (IF) analysis, a sequential staining protocol was employed for the identification of CD4 (1:400 dilution, Proteintech, 67786-1-Ig), CXCR5 (1:400 dilution, Abclonal, A8950) and PD-1 (1:4000 dilution, Proteintech, 66220-1-Ig) markers. Following the identical tissue preparation and antigen retrieval procedures, non-specific binding sites were blocked prior to overnight incubation with primary antibodies at 4 °C. Following extensive washing, species-specific secondary antibodies conjugated with fluorescent dyes were applied. The process of nuclear visualization was achieved through the utilization of DAPI staining, and the slides were preserved using anti-fade mounting medium, with the objective of minimizing fluorescence quenching.

First, the images were captured using a fluorescence microscope. After inputting the image into Image J, we manually adjusted the threshold to precisely separate the positive signals. Then, we measured the percentage of the positive area, which enabled us to achieve quantitative statistics for immunohistochemical staining.

### 2.14 Clinical sample collection

Eighty two post-operation ESCA specimens were obtained from the Department of Thoracic Surgery at the First Affiliated Hospital of Chongqing Medical University. Specimens were collected after obtaining written informed consent, and this study was approved by the Ethics Committee of the First Affiliated Hospital of Chongqing Medical University (2024-075-01).

### 2.15 Statistical analysis

The statistic value of bioinformatics analysis was calculated using t.test in R. The experiment statistic analysis was performed using t.test in Graphpad. The p-value less than 0.05 is considered statistically significant.

## 3 Result

### 3.1 Multi-omics analysis revealing aberrant stemness program activation in ESCA

**Figure 1:**
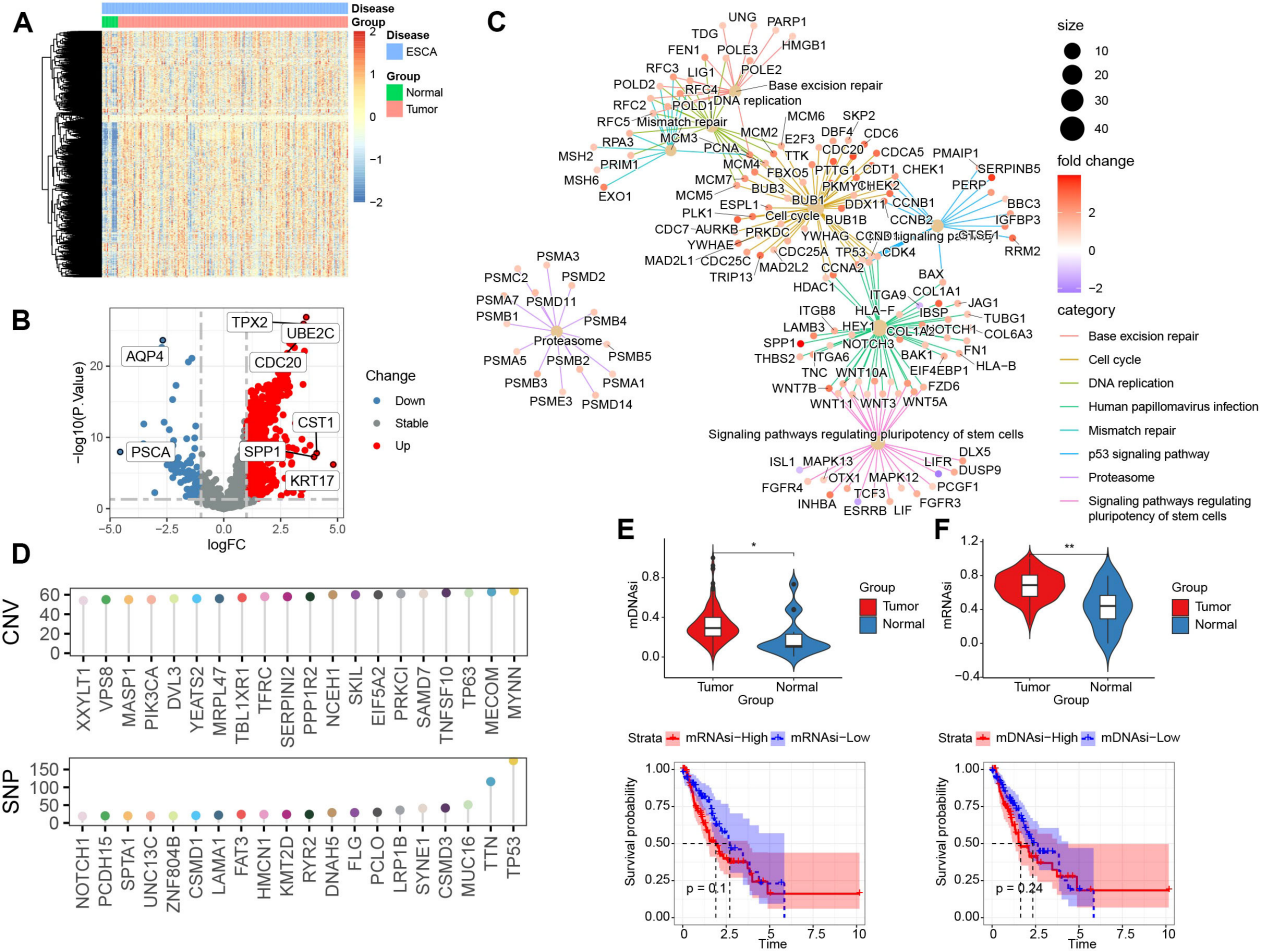
Bulk transcriptomic profiling of gastrointestinal stemness genes and stemness indices in ESCA. **(A)** Heatmap depicting differentially expressed gastrointestinal stemness-related genes between tumors and normal tissues in the TCGA-ESCA cohort. **(B)** volcano plot depicting differentially expressed of gastrointestinal stemness-related genes between tumors and normal tissues in the TCGA-ESCA cohort. **(C)** Pathway enrichment analysis of the stemness gene set performed using clusterProfiler. **(D)** Top 20 genes with the highest alteration frequencies based on CNV and SNP analyses. **(E)** Stemness index quantified by the mRNAsi algorithm in tumor versus normal tissues, showing significantly higher scores in tumors (upper); patients with higher mRNAsi exhibited a trend toward worse survival than those with lower scores (lower). **(F)** Stemness index quantified by the mDNAsi algorithm in tumor versus normal tissues, also demonstrating significantly higher mDNAsi in tumors (upper); higher mDNAsi was associated with a trend toward poorer survival (lower). *p < 0.05, **p < 0.01.

To systematically characterize stemness in ESCA, the heterogeneity of stemness genes (SGs) were first analyzed in bulk RNA-seq data. The result demonstrated distinct SG expression patterns among cancerous growths and surroundings. (**Figure 1A**). And most of these genes were highly expressed in tumor tissues compared with the normal (**Figure 1B**). These stemness-associated genes were predominantly concentrated in pathways involving cell cycle regulation, base excision repair, and DNA repair, consistent with a phenotype characterized by hyperproliferation and matrix remodeling (**Figure 1C**). Stemness genes have been observed to exhibit high levels of both copy number variation (CNV) and single nucleotide polymorphisms (SNP) (**Figure 1D**). Next, we quantified tumor stemness using two established indices, mRNAsi and mDNAsi, which consistently revealed significantly higher stemness iindex in tumor. Patients with higher stemness iindex exhibited a trend toward poorer survival (**Figures 1E, F**). These findings indicate that, in comparison with normal tissue, SGs are significantly dysregulated in ESCA. Such dysregulation has been demonstrated to result in a suboptimal prognosis.

### 3.2 Identifying the malignant-derived stemness gene module

**Figure 2:**
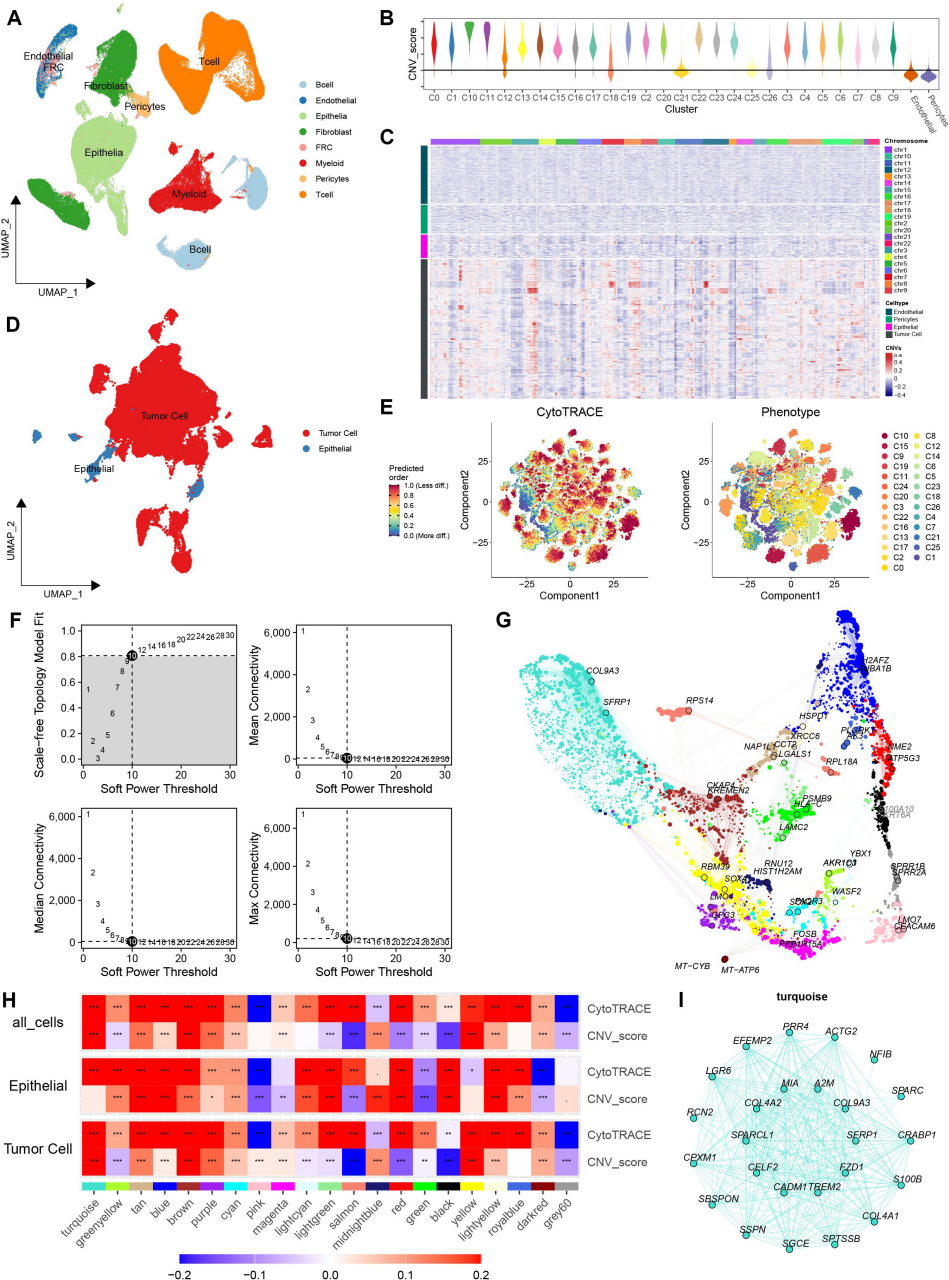
Single-cell identification of stemness-associated malignant subpopulations and hdWGCNA module discovery in ESCA. **(A)** UMAP visualization of major cell populations after dimensionality reduction and clustering of the GSE160269 scRNA-seq dataset. **(B)** CNV scores across cell clusters estimated by inferCNV. **(C)** Heatmap integrating chromosomal CNV profiles, CNV scores, and cell-type annotations, highlighting markedly elevated CNV signals in malignant tumor cells compared with other cell types. **(D)** UMAP visualization of extracted malignant tumor cells and esophageal epithelial cells. **(E)** CytoTRACE-based stemness scoring across single-cell populations. **(F)** Selection of an appropriate soft-thresholding power for hdWGCNA and the corresponding network topology metrics. **(G)** Dimensionality reduction and clustering analysis of hdWGCNA module genes. **(H)** hdWGCNA-derived co-expression network identifying the turquoise module, which is highly associated with tumor-driving programs and stemness maintenance. **(I)** Gene interaction network of stemness-associated candidate genes in the turquoise module.

Stemness genes primarily act on tumor cells. Bulk RNA, however, is unable to identify the cellular origin of genes. The application of single-cell sequencing has been demonstrated to be an effective solution to this issue. We first performed dimensionality reduction and unsupervised clustering of scRNA-seq to generate an ESCA atlas (**Figure 2A**). To identify the malignant cells from the normal epithelial cells, we then performed subcluster analysis of all epithelial cells and assessed copy number variation levels across clusters (**Figure 2B**), enabling the identification of malignant tumor cell populations characterized by prominent CNV signals (**Figures 2C, D**). After further separating tumor cells from epithelial cells, we quantified stemness potential at the single-cell level (**Figure 2E**). To screen core SGs, hdWGCNA analysis was subsequently applied to the tumor cells. After selecting an appropriate soft power (**Figure 2F**), we obtained 21 functional gene modules with distinct expression patterns (**Figure 2G**). And further correlations analysis was calculated between various gene modules and the stemness scores (**Figure 2H**). Interestingly, we found that the turquoise module exhibits a strong correlation with stemness scores. However, this correlation with CNV scores is only evident in tumor cells, demonstrating that the stemness characteristics of this module are relatively specific to tumor cells. Moreover, it had relatively specific category characteristics (**Figure 2I**).

### 3.3 Constructing a stemness prognostic signature

**Figure 3:**
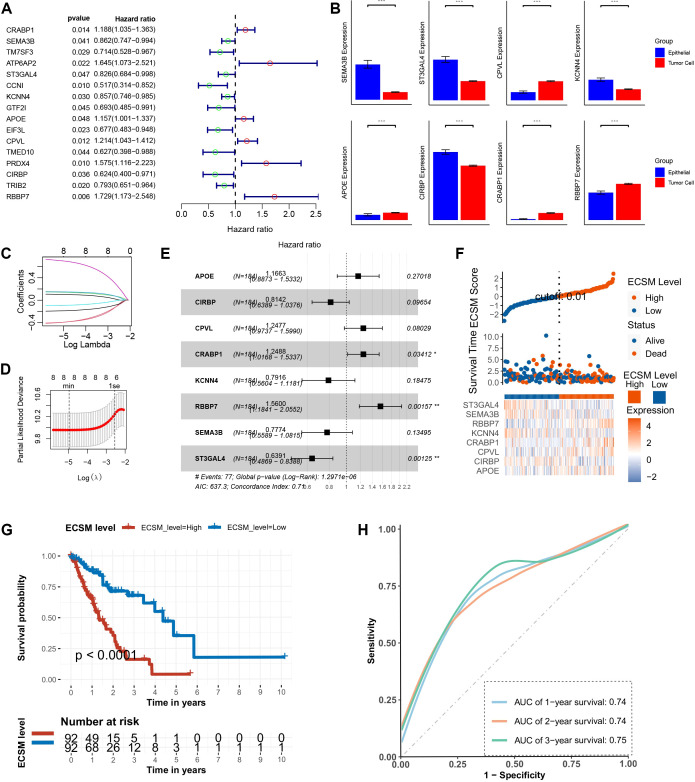
Construction of the ESCA stemness model. **(A)** Univariate Cox regression analysis of genes from the turquoise module to screen candidates significantly associated with patient prognosis. **(B)** Expression validation of prognostic candidates and selection of genes showing marked differential expression between tumors and adjacent normal tissues in scRNA-seq data. **(C)** Machine-learning–based construction of the ESCA stemness model using LASSO regression. **(D)** Mean Square Error curve showing the value of lambda min and lambda 1se. **(E)** Multivariate Cox regression analysis of weighted genes within the ECSM. **(F)** Distribution of ECSM scores across patients. **(G)** Kaplan–Meier survival analysis demonstrating the prognostic stratification performance of the ECSM. **(H)** Time-dependent ROC curves evaluating the predictive accuracy of the ECSM. *p < 0.05, **p < 0.01, ***p < 0.001.

The number of hub genes generated by hdWGCNA is relatively large. Subsequent to this, the hub genes were subjected to further optimization via univariate Cox regression to construct and validate a robust stemness-related prognostic model for ESCA. And 16 SGs influencing prognosis were retained via univariate Cox analysis (**Figure 3A**). Among these, only 8 genes exhibited differences between malignant tumor cells and benign epithelial cells in scRNA-seq dataset (**Figure 3B**). We then applied LASSO regression for further screening (**Figures 3C, D**; **Supplementary Table 3**). Via multivariate Cox regression, we constructed an esophageal carcinoma stemness model (ECSM) to predict the prognosis of ESCA patients (**Figure 3E**). The ECSM effectively stratified patients into distinct prognostic groups, demonstrating favorable predictive performance (**Figure 3F**). Higher ECSM score predicted worse survival (**Figure 3G**). Also, this model performed optimal prediction accuracy in 1-, 2-, and 3-year prognosis (**Figure 3H**). We attempted to validate the model’s efficiency on another dataset. Results showed that the model maintained high predictive efficiency in the validation set, with higher ECSM scores indicating poorer prognosis (**Supplementary Figure 1A**). The model accurately predicted 1-, 2-, and 3-year survival rates (**Supplementary Figure 1B**). Additionally, the model demonstrated consistent predictive efficiency between the validation and experimental sets (**Supplementary Figure 1C**). In the validation set, the model’s predicted values closely approximated actual values (**Supplementary Figures 1D–F**). Subsequently, a pan-cancer analysis was conducted, which revealed that ECSM exhibited efficacy in ESCA and demonstrated promising results across all cancer types (**Supplementary Figure 2**).

### 3.4 The microenvironment infiltration and functional enrichment of ECSM

**Figure 4:**
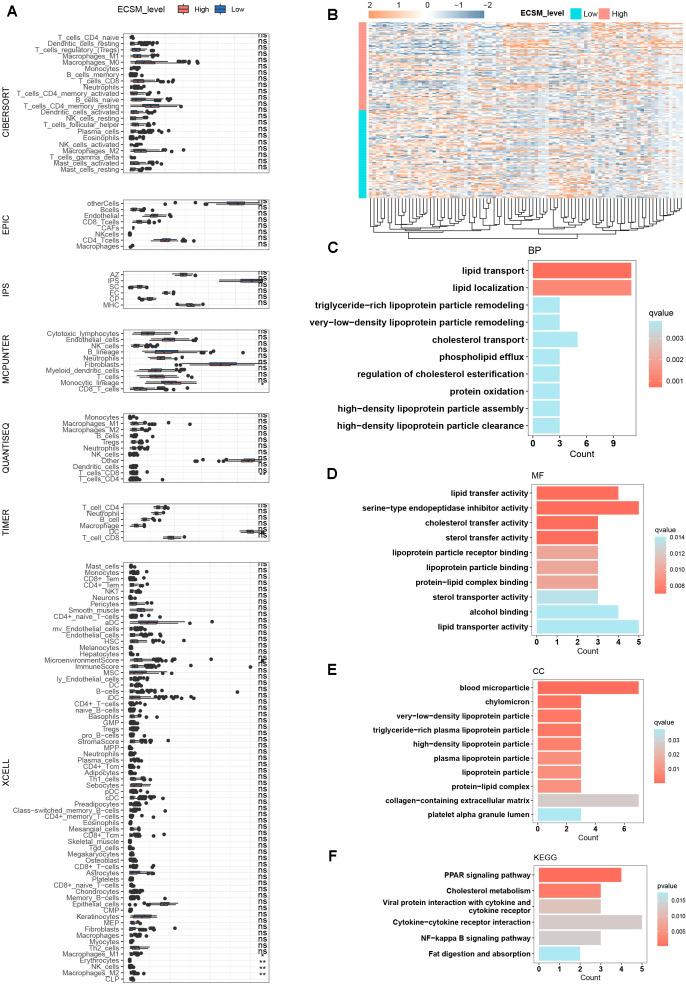
Functional and immune characterization of the ECSM model. **(A)** Immune profiling comparing ECSM-high and ECSM-low groups. **(B)** Heatmap visualization of samples stratified by ECSM scores. **(C–E)** GO enrichment analyses of DEGs, differentially expressed genes between the ECSM-high and ECSM-low groups. **(F)** KEGG pathway enrichment analysis of DEGs between the ECSM-high and ECSM-low groups.

Building on our stemness scoring framework and module gene screening, we identified stemness-associated tumor genes and established ECSM. To further substantiate the stemness-related characteristics of this model, we interrogated the biological and immunological correlates of ECSM. Specifically, ESCA patients were assigned ECSM scores and stratified into ECSM-high and ECSM-low groups, followed by comparative immune profiling. Overall, the two groups exhibited pronounced differences in the tumor immune landscape, with significant alterations in the abundances of multiple immune cell subsets, including macrophages, CD8^+^ T cells, and CD4^+^ T cells, showing the evidence of multiple immune disbalances across different ECSM score groups (**Figure 4A**). We further carried out differential gene expression analysis between the two groups (**Figure 4B**). GO enrichment indicated that these differential expressed genes were predominantly localized to extracellular matrix function as well as blood lipoprotein particles (e.g., chylomicrons, very-low-density lipoproteins, and high-density lipoproteins) and were mainly involved in lipid transport, cholesterol metabolism, and lipoprotein remodeling, with enriched functions related to lipid transfer activity and cholesterol transport (**Figures 4C–E**). Consistently, KEGG analysis revealed significant enrichment of pathways including the PPAR signaling pathway, cholesterol metabolism, cytokine–cytokine receptor interaction, NF-κB signaling, and fat digestion and absorption (**Figure 4F**). Given the established roles of PPAR signaling in metabolic regulation, fatty acid synthesis, and anti-inflammatory responses, and the well-recognized involvement of NF-κB and cytokine–cytokine receptor interaction pathways in tumor progression and immune evasion, these findings collectively support a tight linkage between the ECSM model and stemness-associated tumor programs. The abnormal infiltration of these immune cells and the enrichment of multiple oncogenic pathways provide biological evidence of the prognostic value of ECSM.

### 3.5 Incorporating clinical variables to improve prediction performance and enable nomogram-based individualized risk estimation

**Figure 5:**
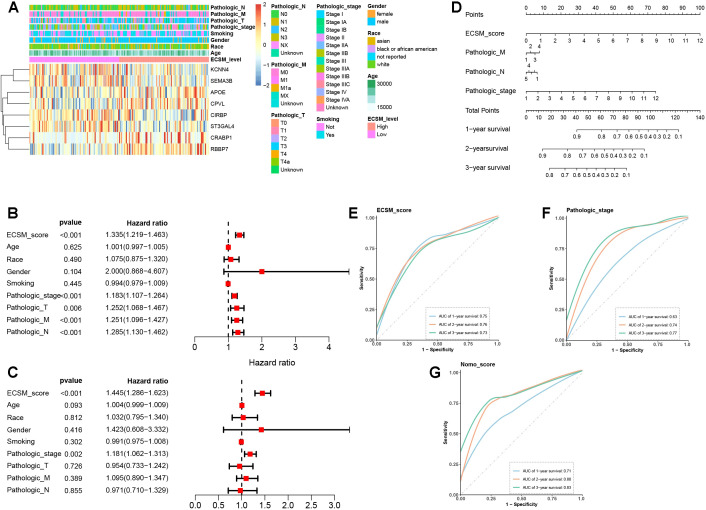
Integration of ECSM with clinical variables and construction of a prognostic nomogram. **(A)** Heatmap showing the distribution of ECSM scores aligned with clinicopathological characteristics in the TCGA-ESCA cohort. **(B)** Univariate Cox regression analysis of ECSM and clinical features. **(C)** Multivariate Cox regression analysis of ECSM and clinical features. **(D)** Nomogram constructed using prognostic factors that remained significant in Cox regression analyses. **(E–G)** Time-dependent ROC curves comparing the predictive performance of different prognostic features.

Finally, to further improve the predictive performance of the ECSM, we aligned ECSM scores with clinicopathological characteristics such as age, gender, race, smoking history, and TMN stage (**Figure 5A**, **Supplementary Table 4**). To minimize the impact of potential confounders, we performed both univariate Cox regression analyses incorporating ECSM and clinical variables to identify prognostic factors. The results demonstrated that ECSM score, pathological stage, pathological stage M, and pathological stage N were significantly meaningful for predicting prognosis (**Figure 5B**). Multivariate Cox analysis demonstrated that the ECSM score holds greater significance than other clinical factors (**Figure 5C**). Also, it was an independent prognostic factor. The variables that remained significant in univariate Cox analysis were then integrated to construct a prognostic nomogram (**Figure 5D**). We subsequently compared the prognostic accuracy of nomogram, ECSM, and clinical factors, which showed that the ECSM showed best performance in predicting 1-year survival (**Figure 5E**). Notably, the nomogram model combining ECSM with clinical features achieved the best overall predictive performance (**Figures 5F, G**). As observed in the Cox analysis, pathologic M and N had inferior predicting abilities (**Supplementary Figures 3A, 3B**).

### 3.6 RBBP7 promoting stemness phenotypes and malignant behavior

**Figure 6:**
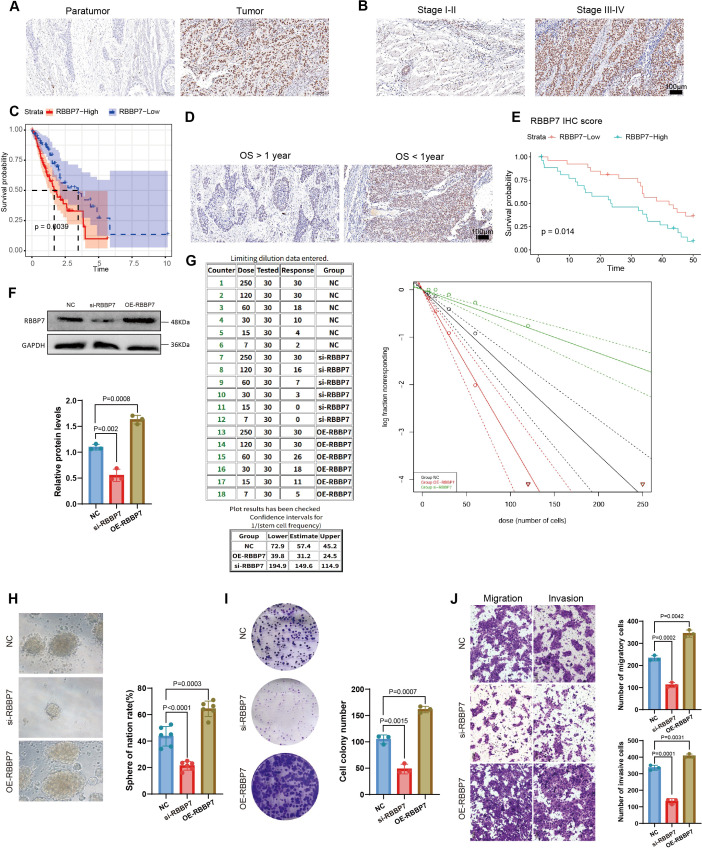
Identification the risk role of RBBP7 and experimental validation of RBBP7. **(A)** Representative IHC, immunohistochemistry images showing lower RBBP7 expression in adjacent normal tissues compared with tumor tissues. **(B)** IHC staining showing increased RBBP7 expression in stage III-IV tumors relative to stage I–II tumors. **(C)** Kaplan–Meier analysis in the TCGA-ESCA cohort confirming significantly worse survival in patients with high RBBP7 expression. **(D)** IHC staining in a clinical cohort showing significantly higher RBBP7 expression in patients with poorer prognosis (<1 year) than in those with better prognosis (≥1 year). **(E)** Kaplan–Meier survival curves based on IHC scores demonstrating prognostic differences across RBBP7 expression groups. **(F)** Western blotting and statistical plot confirming knockdown and overexpression efficiency of RBBP7. **(G)** *In vitro* limiting dilution assay demonstrating stemness phenotype upon RBBP7 knockdown and overexpression (left); the statistical plot of limiting dilution assay (right). **(H)** Tumorsphere formation assay showing sphere size and number. **(I)** Colony formation assay indicating the clonogenic capacity (left); the statistical plot of colony formation assay (right). **(J)** Transwell assays demonstrating cell migration and invasion ability (left); the statistical plot (right).

Among the model genes, RBBP7 had the highest risk coefficient and was identified as an independent adverse prognostic factor (**Figure 3E**). Consistently, immunohistochemical staining confirmed differential RBBP7 expression between tumor and normal tissues (**Figure 6A**), and revealed stage-dependent upregulation of RBBP7 in clinical ESCA specimens, with higher expression observed in more advanced disease (**Figure 6B**). Moreover, the stratification by RBBP7 expression indicated significantly poorer survival in the RBBP7-high group (**Figure 6C**). In an independent clinical cohort, patients were further categorized by prognosis (<1 year vs. ≥1 year), and IHC staining confirmed higher RBBP7 expression in patients with unfavorable outcomes (**Figure 6D**). Kaplan–Meier analysis based on IHC scores further demonstrated significant worse survival rate in RBBP7-high expression group (**Figure 6E**). What’s more, the prognostic role of RBBP7 is relatively specific to ESCA (**Supplementary Figure 4A**), yet RBBP7 exhibits divergent expression patterns across numerous cancer types when compared to healthy tissues (**Supplementary Figure 4B**).

To further investigate the biological function of RBBP7, we obtained serious ESCA cell lines. The RBBP7 mRNA expression was consistently and markedly higher in malignant esophageal cancer cell lines than in HET1A cells (**Supplementary Figure 5A**). This result was consistent in the protein level (**Supplementary Figure 5B**). We selected ECA109, which exhibited the highest RBBP7 expression, for the knockdown (KD) experiments. The siRNA-3 showed best KD efficiency (**Supplementary Figures 5C, D**), and was then used for downstream assays. We also conducted rescue experiments by overexpressing RBBP7 (**Figure 6F**). To functionally validate the role of RBBP7 in maintaining cellular stemness, we performed an *in vitro* limiting dilution assay, which showed a significant reduction in stemness frequency upon RBBP7 knockdown (**Figure 6G**). In parallel, tumorsphere formation assays indicated that RBBP7 depletion markedly decreased both sphere size and number and it could be revised by the rescue experiment (**Figure 6H**). Colony formation assays further confirmed that RBBP7 knockdown significantly reduced clonogenic capacity in ECA109 cells while overexpression of RBBP7 could increase the clonogenic capacity (**Figure 6I**). Finally, silencing RBBP7 significantly impaired the migratory and invasive abilities of ECA109 cells (**Figure 6J**). Overall, the *in vitro* knockdown or overexpression of RBBP7 was found to significantly reduce or increase the stemness phenotype of ESCA cells, thus demonstrating its efficacy as a parameter for evaluating ECSM modification.

### 3.7 The underling mechanisms of RBBP7 regulating stemness

**Figure 7:**
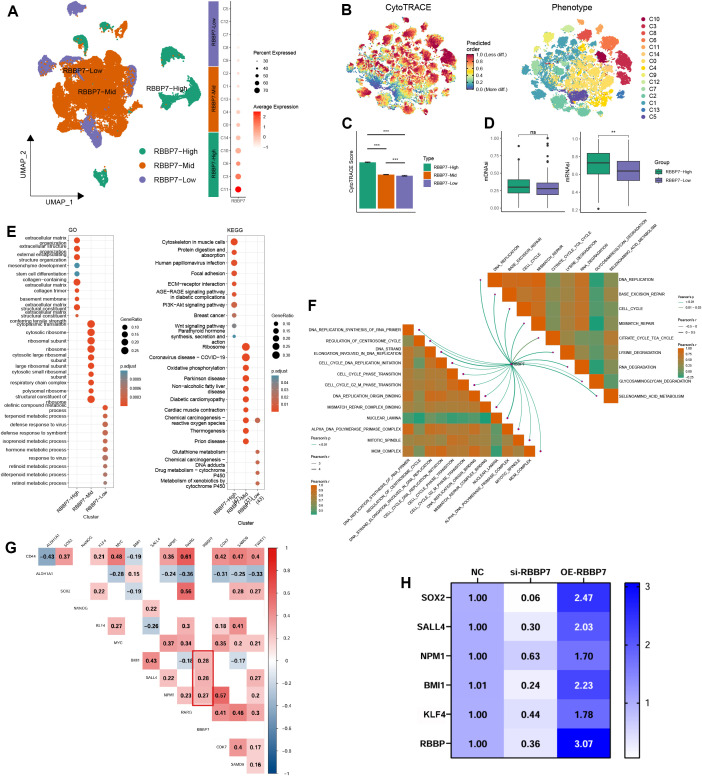
Sequencing and experimental validation of the association between RBBP7 expression and stemness-related programs. **(A)** Re-annotation of malignant tumor cells into three subgroups (RBBP7-high, -intermediate, and -low) based on RBBP7 expression in the scRNA-seq dataset. **(B)** CytoTRACE-based stemness scoring of tumor cells. **(C)** Comparison of CytoTRACE scores across RBBP7-high, -intermediate, and -low groups, showing significant differences among groups. **(D)** Stemness iindex calculated using mRNAsi and mDNAsi, further supporting distinct stemness states between RBBP7-high and RBBP7-low tumor cells. **(E)** GO and KEGG enrichment analyses of subgroup-specific genes identified for each RBBP7 expression group. **(F)** Pathway–pathway correlation heatmap analysis centered on RBBP7-associated signaling programs. **(G)** Correlation heatmap in the TCGA-ESCA cohort depicting associations between RBBP7 and stemness-related genes (e.g., CD44, ALDH1A1, SOX2, KLF4), highlighting significant positive correlations between RBBP7 and BMI1, SALL4, and NPM1. **(H)** Quantitative RT-PCR analysis showing that RBBP7 silencing and overexpression regulated multiple stemness markers, including SOX2, SALL4, NPM1, BMI1, and KLF4, in ECA109 cells.

To identify and biologically validate a key functional gene (RBBP7) from this model, elucidating its role in ESCA stemness and progression, we conducted further analyses of both bulk RNA-seq and scRNA-seq data. Specifically, tumor cells were categorized into three expression levels (high, intermediate, and low) based on RBBP7 presence (**Figure 7A**). We then quantified stemness potential at the single-cell level and aligned CytoTRACE scores with the RBBP7-defined groups (**Figure 7B**). These results demonstrated that an increase in RBBP7 expression leads to a marked increase in the stemness index. (**Figure 7C**). To validate the stemness-related features of RBBP7 from additional perspectives, we applied two independent stemness scoring algorithms in bulk RNA-seq. Consistently, the mRNAsi scores and mDNAsi remained significantly higher in RBBP7-high group (**Figure 7D**). Subsequently, we executed a functional enrichment assessment for delineating the biological mechanisms associated with RBBP7. In the scRNA-seq data, Tumor cells with elevated RBBP7 demonstrated substantial oncogenic signaling and ECM pathway. This demonstrates that RBBP7 plays a role in regulating stemness and acts upon pro-cancer pathways, such as epithelial-mesenchymal transition (**Figure 7E**). Meanwhile, pathway–pathway correlation analysis in the bulk RNA-seq demonstrated that RBBP7 was positively correlated with proliferative programs, including DNA replication, cell-cycle progression, and mismatch repair, further supporting its association with an enhanced proliferative and stemness-like phenotype (**Figure 7F**). What’s more, most of these pathways were risk factors (**Supplementary Table 5**).

To investigate the specific regulatory mechanisms of RBBP7, we next analyzed bulk RNA-seq data to assess correlations between RBBP7 and canonical stemness-associated genes (**Figure 7G**), revealing significant positive correlations between RBBP7 and BMI1, SALL4, and NPM1. Consistently, qRT-PCR analysis of siRBBP7-treated ECA109 cells demonstrated concomitant downregulation of multiple stemness markers, including SOX2, SALL4, NPM1, BMI1, and KLF4. Conversely, RBBP7 overexpression led to an upregulation in the expression levels of these genes (**Figure 7H**). The aforementioned conclusions provide irrefutable evidence that RBBP7 promotes stemness through multiple pathways and regulates stemness markers such as SOX2, SALL4, NPM1, BMI1, and KLF4.

### 3.8 RBBP7 disturbing microenvironment by enhancing T follicular helper cell infiltration

**Figure 8:**
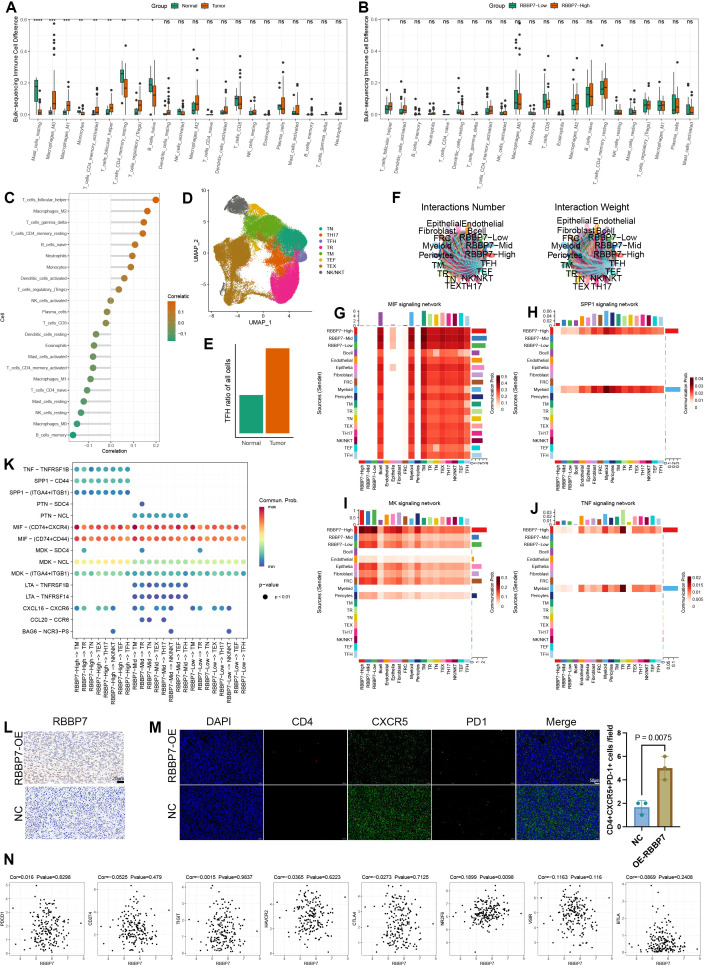
Association of RBBP7 with TFH infiltration and tumor–immune communication in the ESCA microenvironment. **(A)** Immune deconvolution analysis in the TCGA cohort showing distinct immune landscapes between tumor and normal tissues, with significantly higher TFH scores in tumors. **(B)** Comparison of TFH scores between RBBP7-high and RBBP7-low tumors in TCGA, demonstrating significantly elevated TFH infiltration in the RBBP7-high group. **(C)** scRNA-seq–based correlation analysis identifying immune cell subsets associated with RBBP7 expression. **(D)** UMAP visualization of the selected immune cell populations from the scRNA-seq dataset. **(E)** scRNA-seq analysis confirming differential TFH abundance between tumor and normal tissues. **(F)** Predicted interactions numbers and weights by CellChat. **(G–J)** CellChat-inferred ligand–receptor communication patterns across RBBP7 expression groups within the MIF, SPP1, MK, and TNF signaling pathways in the tumor microenvironment. **(K)** Predicted interactions between tumor cells stratified by RBBP7 expression and immune cells. **(L)** IHC staining of RBBP7 in tumor and adjacent tissues from murine ESCA model. **(M)** Multiplex immunofluorescence demonstrating overexpression of RBBP7 increasing TFH cell abundance in murine ESCA tissues (left) and statistical plot (right). **(N)** Correlation analysis between RBBP7 and multiple immune checkpoint genes (PDCD1, CD274, TIGIT, HAVCR2, CTLA4, NRP2, VSIR, and BTLA).

Enhanced tumor stemness is typically associated with tumor progression, processes that are tightly intertwined with the tumor immune microenvironment. To explore potential mechanisms by which RBBP7 sustains stemness, we first performed immune deconvolution analyses in the bulk cohort and confirmed substantial immune-contexture differences between tumor and normal tissues, with significantly higher T follicular helper (TFH) cells in tumors (**Figure 8A**). Stratification by RBBP7 expression further revealed that TFH infiltration was significantly elevated in the RBBP7-high tumors compared with the RBBP7-low group (**Figure 8B**). And TFH cells as the immune subset most strongly and positively correlated with RBBP7 (**Figure 8C**). We performed single-cell subset analysis of T cells (**Figure 8D**). Similarly, TFH cells are far more numerous in tumor tissue than in normal tissue (**Figure 8E**). And we first hypothesize that RBBP7 promotes TFH cell infiltration in tumors and then interrogated cell–cell communication between tumor cells stratified by RBBP7 expression and immune cells (**Figure 8F**). The CellChat analysis suggested that RBBP7-high malignant cells markedly amplified several signaling axes within the tumor microenvironment, including TNF–TNFRSF1B, SPP1–CD44, and SPP1–(ITGA4+ITGB1) (**Figures 8G–J**). It has also been demonstrated that these axes were maintained in communications between T cells and RBBP7-high tumor cells (**Figure 8K**). To validate these computational observations, we established a murine ESCA model (**Figure 8L**). RBBP7 overexpression tumors showed a more significant infiltration of TFH cells (**Figure 8M**). Finally, correlation analyses between RBBP7 and immune checkpoint genes revealed that NR2F6 was statistically significant correlated with RBBP7 (**Figure 8N**). These findings indicate that RBBP7 expression was significantly correlated with increased infiltration of T follicular helper cells and an immunosuppressive microenvironment.

### 3.9 RBBP7 leading targeted-therapy and chemotherapy resistance

**Figure 9:**
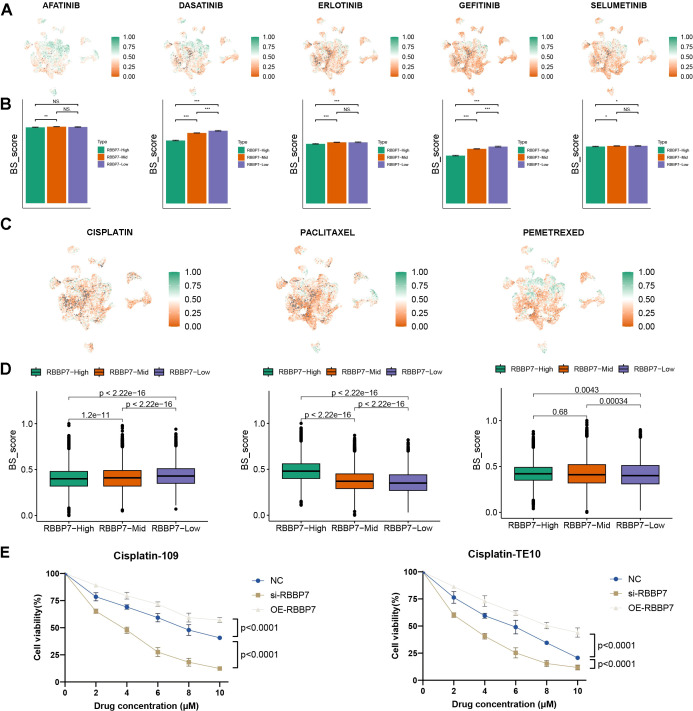
BeyondCell-based single-cell drug response prediction across RBBP7 expression subgroups and validation. **(A)** BeyondCell scoring for targeted agents in tumor cells stratified into RBBP7-high, -intermediate, and -low groups based on scRNA-seq data. **(B)** The statistical plot of BeyondCell scores of targeted agents. **(C)** BeyondCell-based prediction of responses to chemotherapeutic agents across the same RBBP7 expression-defined subgroups. **(D)** The statistical plot of BeyondCell scores of chemotherapeutic agents. **(E)** The cell viability assay of cisplatin treatment.

Previous results have shown that elevated levels of RBBP7 in tumors are associated with increased TFH infiltration, and increased TFH infiltration often predicts better tumor response to neoadjuvant immunochemotherapy (20). Therefore, we hypothesize that RBBP7 may be associated with improved neoadjuvant immunochemotherapy outcomes. However, the current clinical treatment of esophageal cancer primarily relies on chemotherapy, with some patients also receiving targeted therapy; yet, the relationship between RBBP7 and the therapeutic efficacy of these drugs remains unclear. To explore these relationships, we leveraged the drug response scoring framework to predict sensitivity to both targeted agents and chemotherapeutics. A higher BeyondCell (BS) score indicates greater drug sensitivity. Notably, for targeted therapies including dasatinib and gefitinib, BS scores differed significantly among the three RBBP7 expression groups, with the RBBP7-high subset exhibiting a stronger sensitivity profile (**Figures 9A, B**). Of the commonly used chemotherapeutic agents, tumors with high levels of RBBP7 expression are less responsive to cisplatin but more responsive to paclitaxel and pemetrexed (**Figures 9C, D**). We conducted *in vitro* experiments using the 109 and TE10 esophageal cancer cell lines and found that RBBP7 overexpression significantly reduced the cells’ sensitivity to cisplatin (**Figure 9E**). In contrast, the cells’ sensitivity to paclitaxel and pemetrexed was only marginally affected by RBBP7 expression levels (**Supplementary Figures 6A, B**). Furthermore, the BS score for cisplatin was negatively correlated with the IC50 values obtained from the cell experiments (**Supplementary Figure 6C**). The varying responsiveness of RBBP7 to different drugs guides decisions on the clinical utilization of drugs.

## 4 Discussion

Esophageal cancer is a malignant tumor of the digestive system whose incidence and mortality have continued to increase worldwide, substantially compromising patients’ quality of life and imposing a considerable socioeconomic burden on healthcare systems (21). Early-stage disease is frequently asymptomatic, leading to delayed diagnosis and advanced-stage presentation in many patients. Moreover, the lack of well-defined molecular subtypes in esophageal cancer has hindered the implementation of truly individualized therapeutic strategies. Despite advances in surgery and multimodal treatment, the 5-year overall survival (OS) remains unsatisfactory, approximately 30–40% (22). For patients receiving chemotherapy, the median progression-free survival is only 4–6 months and the median OS is approximately 9–12 months (21, 23). These clinical challenges underscore an urgent need to elucidate the molecular basis of esophageal cancer and to identify actionable targets.

Tumor stemness is increasingly recognized as a key driver of aggressiveness, therapeutic resistance, and recurrence, and stemness-associated genes represent an emerging frontier in precision oncology. In this study, we developed an Esophageal carcinoma stemness model (ECSM) that effectively predicted survival probability in ESCA. Furthermore, by integrating ECSM with clinical variables, we constructed a nomogram incorporating the ECSM score, which demonstrated satisfactory predictive performance. Currently, many studies use bulk RNA-seq data to create prognostic models (20, 24). However, as these models incorporate genes from differential expression analyses of bulk RNA-seq data, it is impossible to ascertain the cellular origin of these genes. Our ECSM model, on the other hand, first screens for stemness genes based on cell-type-specific expression and differential expression patterns. This approach better captures the defining characteristics of stemness. Unlike some models that only reflect one-year survival rates, the ECSM model can predict survival rates at one, two and three years, offering greater applicability (25). Compared to other models, the ECSM model demonstrates a significant advantage in terms of accuracy (24). Collectively, these results suggest that ECSM may serve as a reliable model for predicting survival outcomes and stemness intensity in ESCA, thereby providing a rationale for the development of novel therapeutic strategies.

By interrogating the ECSM signature, we identified retinoblastoma-binding protein 7 (RBBP7) as the most prominent high-risk gene. Our findings indicate that elevated RBBP7 expression is associated with unfavorable survival in esophageal cancer; specifically, patients with high RBBP7 expression exhibited a significantly shorter median survival than those with low expression. This observation is consistent with prior reports and reinforces the potential of RBBP7 as a prognostic biomarker (4, 26). Clinically, assessment of RBBP7 expression may aid in refining risk stratification and optimizing personalized treatment decisions, with the goal of improving survival outcomes (27).

Notably, the expression dynamics of RBBP7 across disease states also provide clinically relevant insights. We observed that RBBP7 expression was significantly higher in tumor tissues than in normal controls and was positively correlated with stemness scores, supporting a potential role in early diagnosis, disease monitoring, and stemness maintenance (2, 27). When combined with other biomarkers, RBBP7 may serve as an auxiliary indicator to enhance diagnostic accuracy and to improve therapeutic effectiveness in esophageal cancer (28).

T follicular helper (TFH) cells represent a specialized subset of CD4^+^ T cells enriched in lymphoid follicles. Previous studies suggest that persistent TFH responses may promote uncontrolled epithelial proliferation via the IL-21/STAT3 axis and facilitate malignant transformation in the context of chronic inflammation. In addition, TFH-driven expansion of regulatory B cells (Bregs) and subsequent IL-10 production can suppress effector T-cell activity, thereby contributing to immune evasion. Building upon these observations, our cell–cell communication analyses revealed significant interactions between RBBP7-associated tumor programs and TFH cells, implying that RBBP7 may influence tumor stemness, at least in part, by modulating the immune microenvironment (29, 30). This finding provides a potential mechanistic basis for future immunotherapeutic strategies and may help improve therapeutic responsiveness and prognosis in patients with esophageal cancer (31).

Consistent with our computational analyses, *in vitro* experiments further demonstrated that RBBP7 knockdown markedly inhibited proliferation and migration of ESCA cells and concurrently reduced the expression of multiple stemness markers. Moreover, limiting dilution and sphere-formation assays confirmed that silencing RBBP7 significantly diminished stemness-associated properties, highlighting an essential role of RBBP7 in sustaining malignant growth. These results provide experimental support for the development of RBBP7-targeted therapeutic approaches (32, 33). In combination with existing modalities, targeting RBBP7 may enhance treatment efficacy and offer new therapeutic options for esophageal cancer patients (30).

Several limitations should be acknowledged. First, the sample size was relatively limited, and multi-center clinical validation was not performed, which may affect the generalizability of our conclusions. Second, potential batch effects among public datasets may influence the estimation of RBBP7 expression levels and the robustness of its prognostic value. And in the section on drug sensitivity validation, we did not take into account the effects of the immune microenvironment. Although our integrative bioinformatics analyses and experimental validation collectively support the biomarker potential of RBBP7, future studies with larger, multi-center cohorts are warranted to validate these findings and to further delineate the mechanistic role of RBBP7 in ESCA.

In summary, this study highlights the prognostic relevance of the stemness-associated gene RBBP7 in esophageal cancer and supports its potential utility as a biomarker for risk stratification and personalized management. Further mechanistic investigations of RBBP7 and its broader oncogenic impact may open new avenues for targeted therapy development, ultimately improving survival and quality of life for patients with esophageal cancer.

## Data Availability

The original contributions presented in the study are included in the article/**Supplementary Material**. Further inquiries can be directed to the corresponding authors.
