# Supplementary material for: Stemness signature RBBP7 reprograms the immune microenvironment to inform a prognostic model in esophageal carcinoma
Source: Front Immunol. 2026 May 19;17:1789900. doi: 10.3389/fimmu.2026.1789900 (PMC13226502; doi:10.3389/fimmu.2026.1789900)
Supplement: Supplementary file 1 [file DataSheet1.docx]

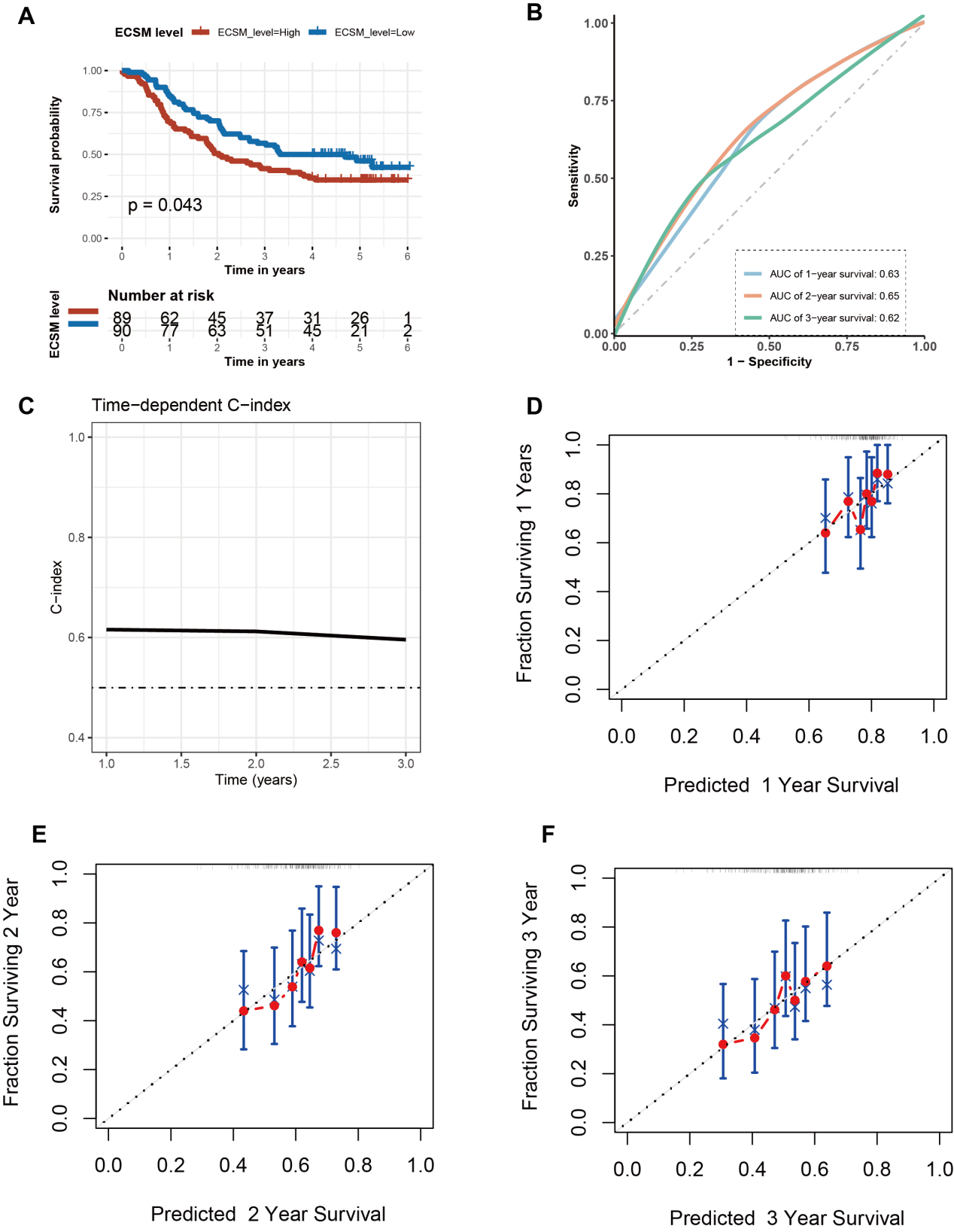


**Supplementary Figure 1.** Validation the prognostic efficiency of ESCM. (A) The KM plot showing survival probability difference of ESCM-high and ESCM-low groups. (B) The ROC curve showing the prediction accuracy of ESCM in validation set. (C) The C-index plot showing the consistency of predictions. (D) The calibration curve of 1-year survival in validation set. (E) The calibration curve of 2-year survival in validation set. (F) The calibration curve of 3-year survival in validation set.


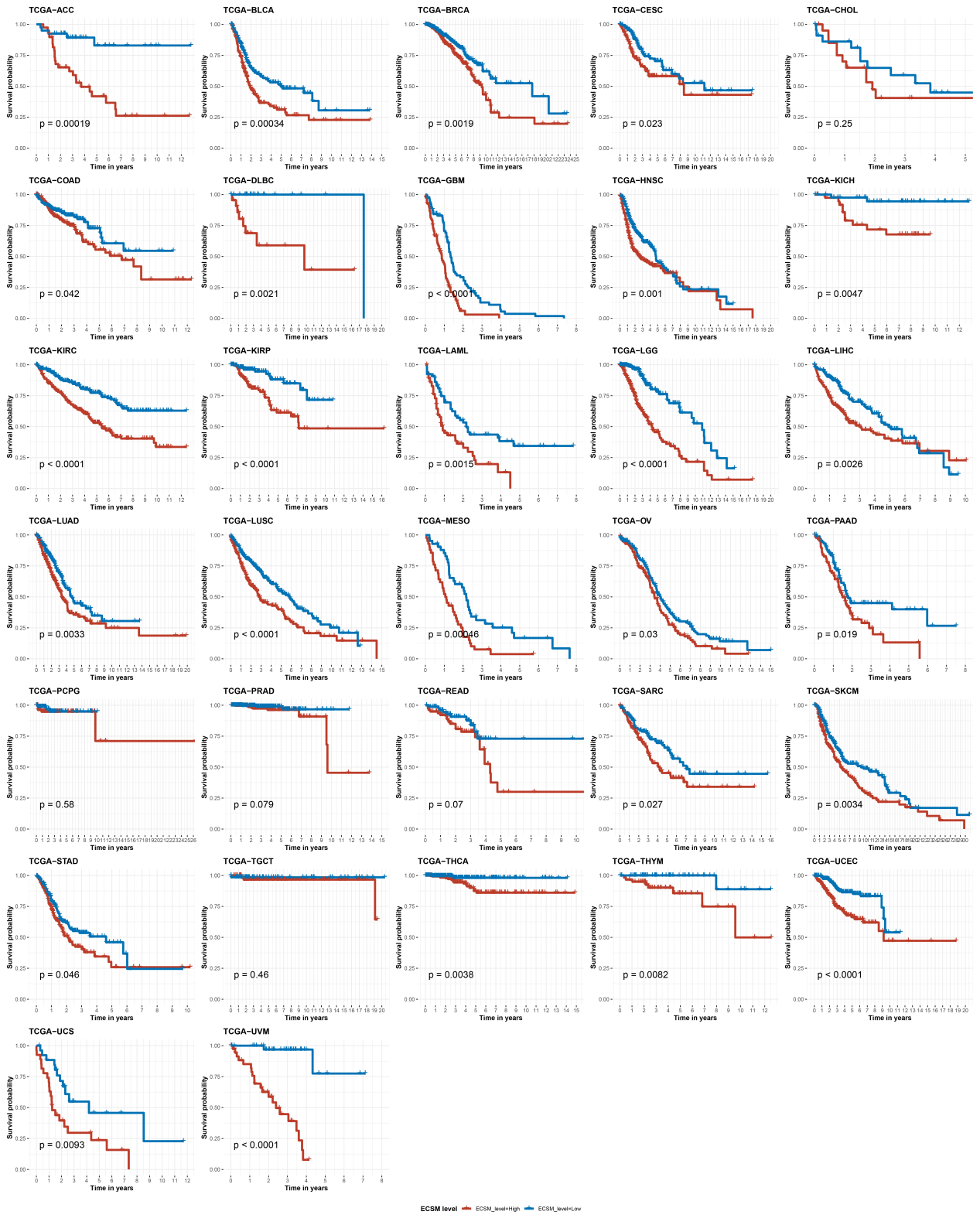


**Supplementary Figure 2.** The pan-cancer prognosis predicting efficiency of ESCM.
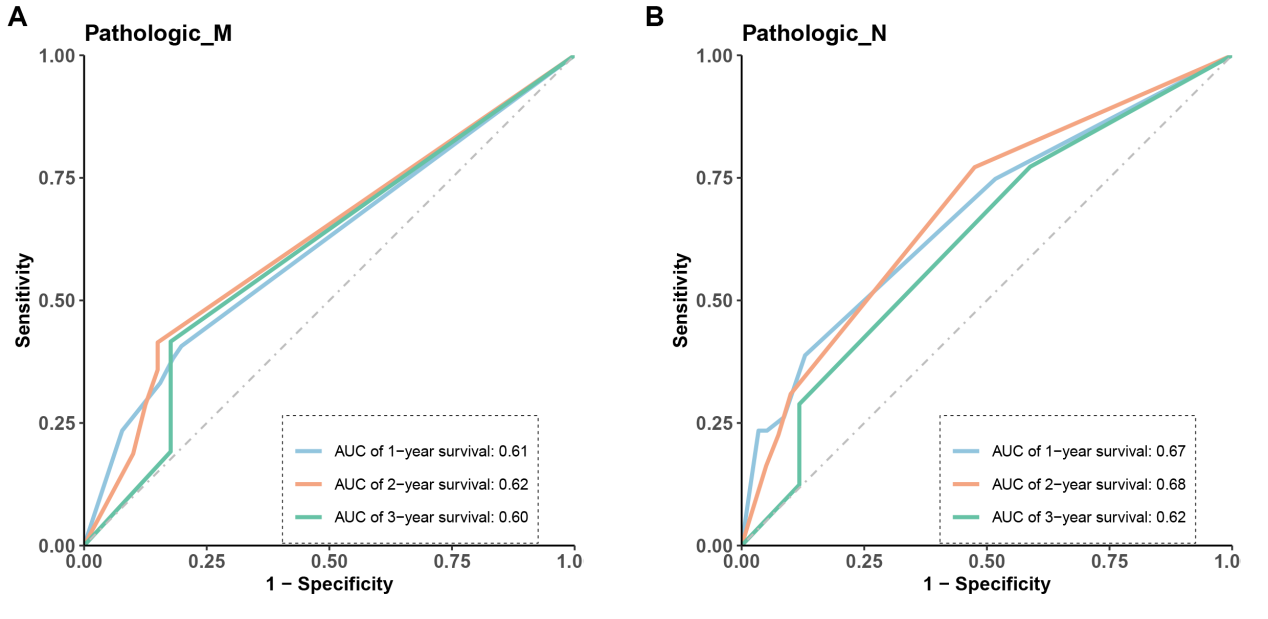


**Supplementary Figure 3.** The ROC curve of pathologic M and N. (A) The ROC curve of pathologic M in survival prediction. (B) The ROC curve of pathologic N in survival prediction.


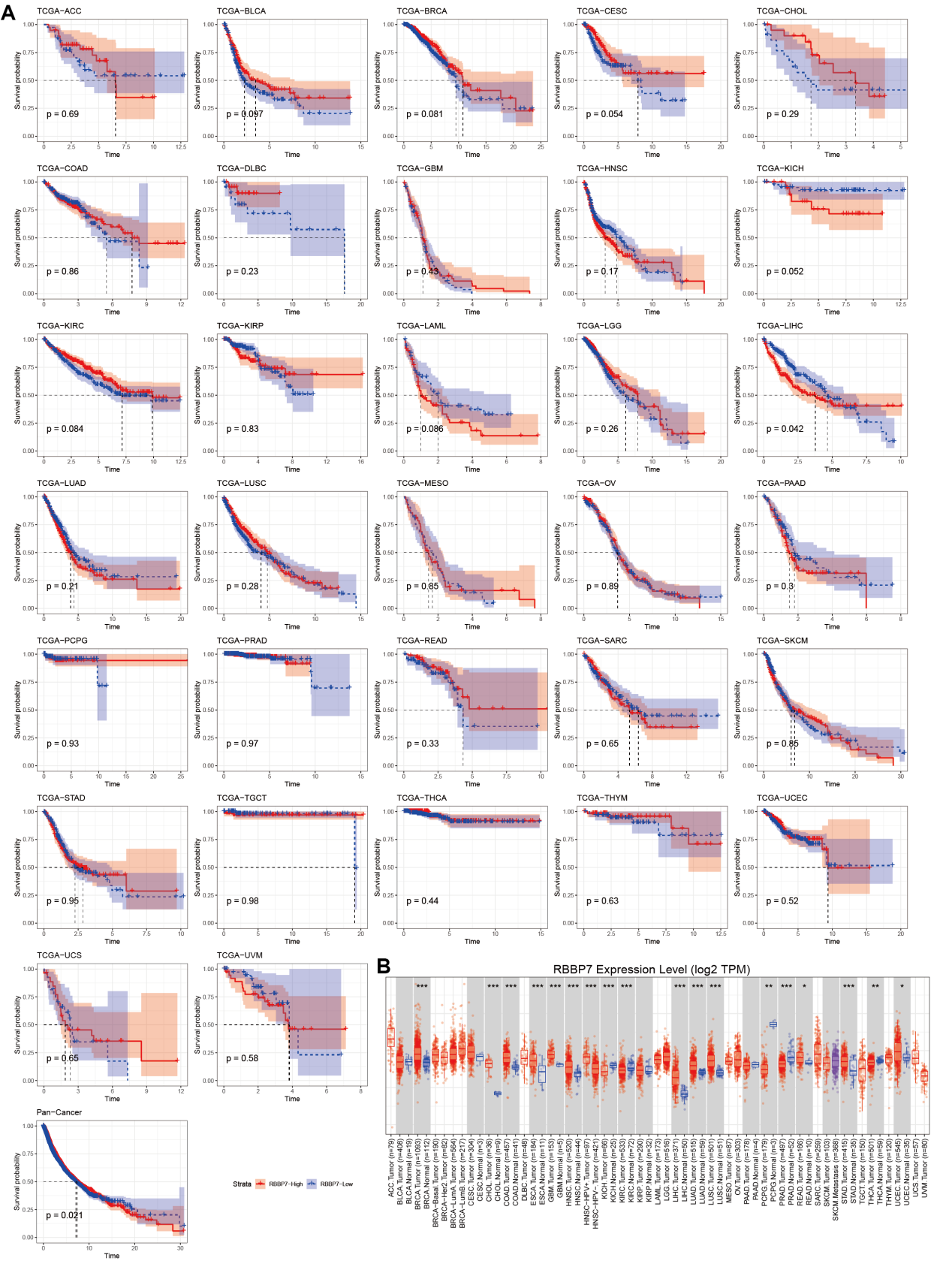
**Supplementary Figure 4.** The pan-cancer analysis of RBBP7. (A) The KM curve showing the survival difference of RBBP7-high and RBBP7-low. (B) The pan-cancer expression difference of RBBP7 in tumor and normal.


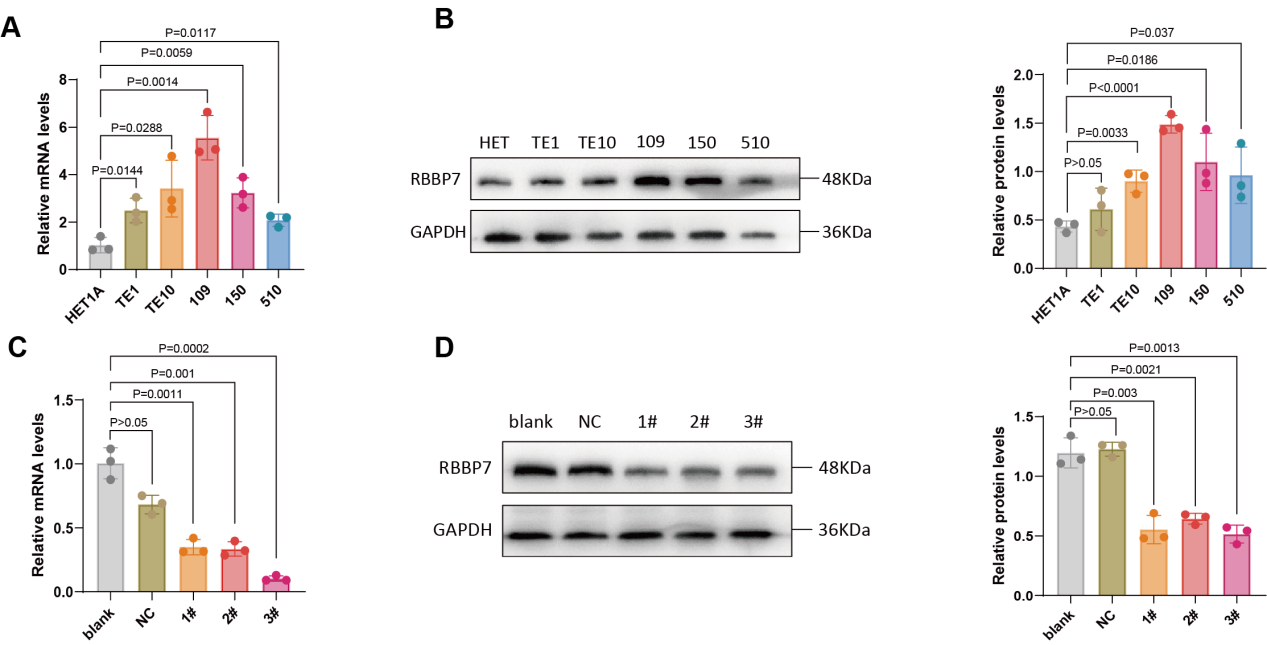


**Supplementary Figure 5.** Experimental validation of RBBP7 expression in vitro. (A) Quantitative RT-PCR analysis showing higher RBBP7 expression in ESCA cell lines than in the normal esophageal epithelial cell line HET1A. (B) Western blotting confirming elevated RBBP7 protein expression in ESCA cell lines relative to HET1A, with ECA109 selected as the RBBP7-high cell line for subsequent experiments (left); the statistical bar chart of western blot (right). (C) Quantitative RT-PCR validating the knockdown efficiency of siRBBP7 in ECA109 cells. (D) Western blotting validating the knockdown efficiency of siRBBP7 in ECA109 cells (left); the statistical bar chart of western blot (right).


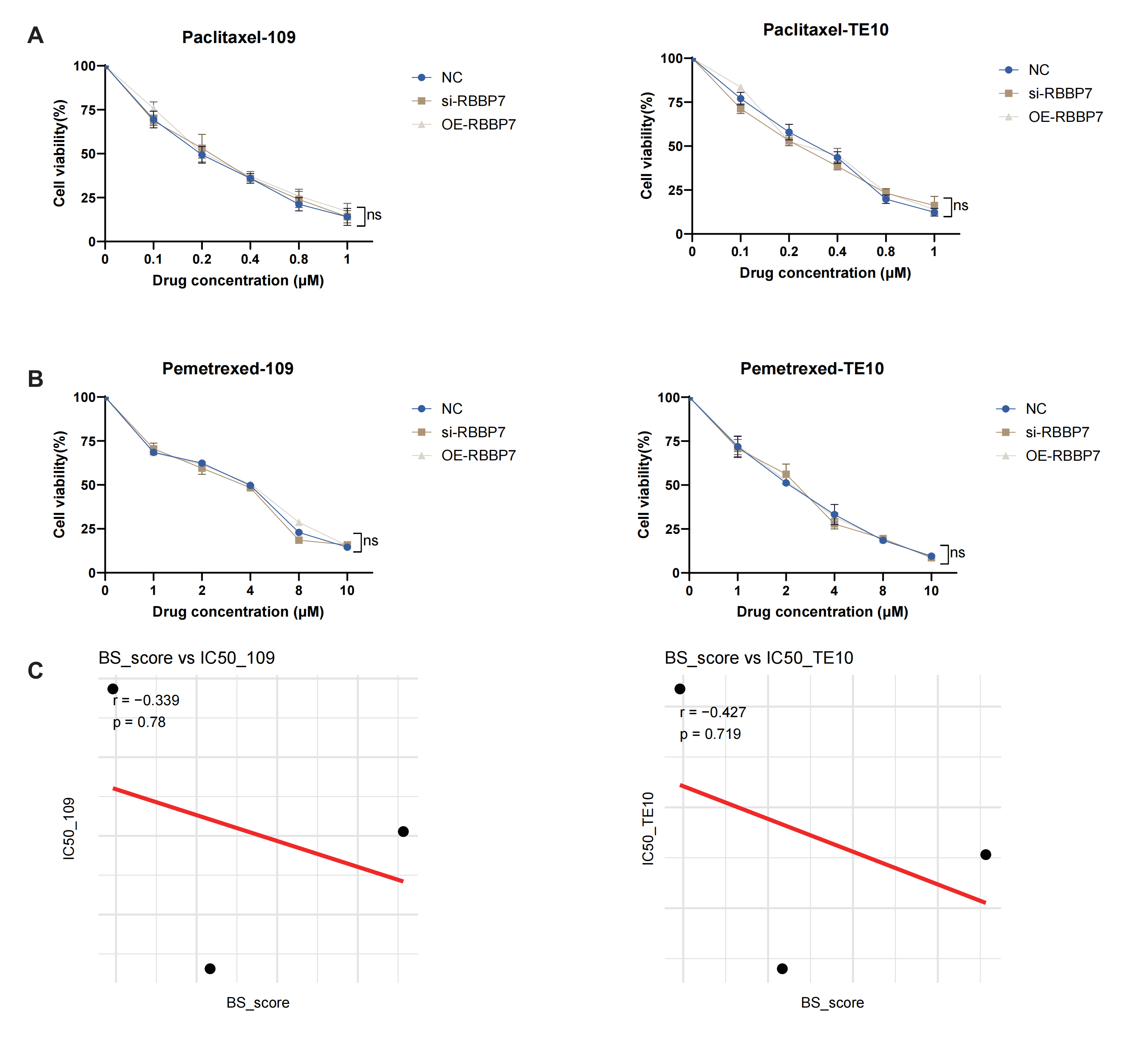


**Supplementary Figure 6.** Experimental validation of the correlation between RBBP7 expression and cell viability in chemotherapy. (A) Dot plot showing cell viability in 109 and TE10 treated with paclitaxel. (B) Dot plot showing cell viability in 109 and TE10 treated with pemetrexed. (C) Dot plot showing correlation between BeyondCell score and IC50 treated with cisplatin.
